# Longitudinal study of childhood sleep trajectories and adolescent mental health problems

**DOI:** 10.1093/sleepadvances/zpae013

**Published:** 2024-03-07

**Authors:** Evelyne Touchette, Gabrielle Fréchette-Boilard, Dominique Petit, Marie-Claude Geoffroy, Marie-Hélène Pennestri, Sylvana Côté, Richard E Tremblay, Amélie Petitclerc, Michel Boivin, Jacques Montplaisir

**Affiliations:** Department of Psychoeducation, Université du Québec à Trois-Rivières, Québec city, Québec, Canada; Research Unit on Children’s Psychosocial Maladjustment, Québec, Canada; Center for Advanced Research in Sleep Medicine, Hôpital du Sacré-Cœur de Montreal, Centre intégré universitaire de santé et de services sociaux du Nord-de-L’île-de-Montréal, Montréal, Québec, Canada; Department of Psychoeducation, Université du Québec à Trois-Rivières, Québec city, Québec, Canada; Center for Advanced Research in Sleep Medicine, Hôpital du Sacré-Cœur de Montreal, Centre intégré universitaire de santé et de services sociaux du Nord-de-L’île-de-Montréal, Montréal, Québec, Canada; Department of Psychiatry, University of Montreal, Montréal, Québec, Canada; Research Unit on Children’s Psychosocial Maladjustment, Québec, Canada; Department of Psychiatry, McGill University, Montréal, Québec, Canada; Research Unit on Children’s Psychosocial Maladjustment, Québec, Canada; Center for Advanced Research in Sleep Medicine, Hôpital du Sacré-Cœur de Montreal, Centre intégré universitaire de santé et de services sociaux du Nord-de-L’île-de-Montréal, Montréal, Québec, Canada; Department of Educational and Counselling Psychology, McGill University, Montréal, Québec, Canada; Hôpital en Santé Mentale Rivière-des-Prairies, Centre intégré universitaire de santé et de services sociaux du Nord-de-L’île-de-Montréal, Montréal, QC, Canada; Research Unit on Children’s Psychosocial Maladjustment, Québec, Canada; Department of Social and Preventive Medicine, University of Montreal, Montréal, QC, Canada; Research Unit on Children’s Psychosocial Maladjustment, Québec, Canada; School of Public Health, Physiotherapy and Population Science, University College Dublin, Dublin, Ireland; Research Unit on Children’s Psychosocial Maladjustment, Québec, Canada; Department of Psychology, Laval University, Québec city, Québec, Canada; Research Unit on Children’s Psychosocial Maladjustment, Québec, Canada; Department of Psychology, Laval University, Québec city, Québec, Canada; Center for Advanced Research in Sleep Medicine, Hôpital du Sacré-Cœur de Montreal, Centre intégré universitaire de santé et de services sociaux du Nord-de-L’île-de-Montréal, Montréal, Québec, Canada; Department of Psychiatry, University of Montreal, Montréal, Québec, Canada

**Keywords:** childhood sleep, psychopathology, adolescence, longitudinal study

## Abstract

**Study Objective:**

To investigate whether childhood sleep trajectories are associated with mental health symptoms such as social phobia, generalized anxiety, depression, attention deficit hyperactivity disorder (ADHD), conduct problems, and opposition at age 15.

**Methods:**

A total of 2120 children took part in the Quebec Longitudinal Study of Child Development. Childhood sleep trajectories were computed from maternal reports at 2.5, 3.5, 4, 6, 8, 10, and/or 12 years. At age 15, 1446 adolescents filled out mental health and sleep questions. A path analysis model was assessed in the full sample.

**Results:**

Four childhood nocturnal sleep duration trajectories were identified: (1) a short pattern (7.5%), (2) a short-increasing pattern (5.8%), (3) a 10 hours pattern (50.7%), and (4) an 11 hours pattern (36.0%). Three childhood sleep latency trajectories were found: (1) a short pattern (31.7%), (2) an intermediate pattern (59.9%), and (3) a long pattern (8.4%). Finally, two childhood wakefulness after sleep-onset trajectories were found: (1) a normative pattern (73.0%) and (2) a long pattern (27.0%). The path analysis model indicated that children following a long childhood sleep latency trajectory were more likely to experience symptoms of depression (*β* = 0.06, 95% CI: 0.01 to 0.12), ADHD (*β* = 0.07, 95% CI: 0.02 to 0.13), conduct problems (*β* = 0.05, 95% CI: 0.00 to 0.10) and opposition (*β* = 0.08, 95% CI: 0.02 to 0.13) at age 15.

**Conclusions:**

This longitudinal study revealed that children presenting a long sleep latency throughout childhood are at greater risk of symptoms of depression, ADHD, conduct problems, and opposition in adolescence.

**Statement of Significance**

Mental health professionals should promote treating difficulties in initiating sleep throughout childhood to nurture mental health later on in adolescence.

## Introduction

Up to 20% of adolescents worldwide report mental health problems [1]. A large-scale meta-analysis of 192 epidemiological studies reported that the peak age for presenting the greatest proportion of any mental health problems in adolescence was 14.5 years [2]. In turn, mental health problems have a great impact on early adult functioning [3]. Sleep plays a vital role in maintaining sound mental health across the lifespan. For instance, a cross-sectional study from the Canadian Health Survey on Children and Youth revealed that young individuals with poor sleep were more likely to have a lower general mental health index, psychosocial difficulties, such as mood/anxiety/attention disorder and have required mental health care in past year [4]. Furthermore, a Norwegian survey of adolescents (2018) showed that, independently of sociodemographic background variables, different sleep dimensions (e.g. sleep quantity, sleep quality, and daytime sleepiness) of adolescents were associated with concurrent mental health problems such as emotional problems, conduct problems, and attention deficit hyperactivity disorder (ADHD) [5].

Sleep problems co-occur with or can precede mental health problems early in development. Childhood sleep problems are quite common, with a prevalence between 25% and 40% [6, 7]. They usually present in the form of sleep-onset difficulties/bed resistance and frequent or long awakenings. These two problems have been termed protodyssomnias [8] because they often lead, respectively, to sleep initiation and sleep maintenance insomnia if not corrected. In childhood, difficulties in initiating sleep or bedtime resistance are defined by a latency to fall asleep greater than 30 minutes [8]. Difficulties in maintaining sleep are defined as awakenings of about 5 minutes or more after sleep onset, happening twice or more per night [8]. Both problems will likely affect sleep duration. Many studies found bidirectional associations between short sleep duration or childhood sleep problems (but not sleep efficiency [9]) and internalizing and externalizing problems before adolescence [9–11]. More importantly, a systematic review [12] of seven longitudinal and prospective studies observed a possible causal relationship between early childhood sleep disorders; mainly trouble falling asleep, insomnia, and short sleep duration; and three mental health problems in adolescence, namely anxiety, depression, and ADHD. However, traditional externalizing problems and other mental health disorders, such as social phobia, generalized anxiety, conduct disorder, and opposition, were not investigated in the papers included in the review. Yet these disorders are prevalent in adolescence [13] and could also be related to childhood sleep problems.

Considering the importance of investigating both internalizing and externalizing mental health problems during adolescence as a function of the various childhood sleep dimensions [14], the present prospective and longitudinal study will investigate difficulties in initiating sleep, wake after sleep onset (WASO), and nighttime sleep duration during childhood in relation to a large spectrum of adolescent DSM-5-defined mental health symptoms, namely social phobia, generalized anxiety, depressive, ADHD, conduct problems. and opposition at age 15. The use of childhood trajectories for sleep variables will add robustness to the analyses. The study will also control for the following covariates: sociodemographic variables, early childhood behavioral problems, and concomitant sleep dimensions. Based on aforementioned literature, we expect that children with short-persistent nocturnal sleep duration, a long sleep latency, and a long WASO will be associated positively with mental health outcomes at age 15 after adjusting for covariates.

## Materials and Methods

### Setting, participants, and procedure

This study is part of the initial cohort of Quebec Longitudinal Study of Child Development, which is conducted by the Institut de la statistique du Québec. This sample is representative of children born in 1997–1998 in the province of Quebec, Canada, excluding children living in Cree or Inuit territories, Indian reserves, and northern Quebec. All children were recruited through the Quebec Master Birth Registry stratified based on living area and birth rate. Before participating in the study, all families received detailed information by mail on the aims and procedures of the research program and signed a consent form. Families were included if the pregnancy had lasted between 24 and 42 weeks, and the mother could speak French or English. A total of 2120 children participated in the first data collection when they were aged 5 months. At age 15, 1446 adolescents (65.0% of the initial sample) filled out a self-reported questionnaire on mental health. For more information, visit the website of cohort’s profile [15]: https://www.jesuisjeserai.stat.gouv.qc.ca/a_propos/etude_phase3_an.html.

### Childhood sleep dimensions

*Nighttime sleep duration* was measured by maternal reports at 2.5, 3.5, 4, 6, 8, 10, and 12 years of age by the following open question: “Indicate how long in total your child sleeps during the night (on average). Do not count the hours that your child is awake.” *Sleep latency* was measured by maternal reports at 2.5, 3.5, 4, 6, 8, and 10 years of age by a Likert question: “How long does your child take to fall asleep?” Response categories were: (1) < than 15 minutes, (2) from 15 minutes to < than 30 minutes, (3) from 30 minutes to < 45 minutes, (4) from 45 minutes to < than 60 minutes, and (5) ≥ 60 minutes. *Wakefulness after sleep onset (WASO)* was measured by maternal reports at 2.5, 3.5, 4, 6, 8, and 10 years of age by an open question-ended: “Indicate the total duration of time spent awake at night (on average).”

### Adolescent mental health problems at age 15

Adolescents completed the *Mental Health and Social Inadaptation Assessment* (MIA) at age 15 [16]. Using a dimensional approach, this self-reported questionnaire contains 113 items rated on a 3-point Likert scale (1 = never, 2 = sometimes, and 3 = often) and takes 20–25 minutes to fill out. It has been designed to assess the presence of mental health symptoms over the past 12 months [17]. Among internalizing mental health problems, we retained those with “excellent” (≥0.90) or “good” (≥80 to < 90) Cronbach’s alphas (*α*) and diagnoses that were included in the Diagnostic and Statistical Manual of Mental Disorders (DSM) of the 5th edition: *social phobia* (8 items, *α* = 0.90), *generalized anxiety* (9 items, *α* = 0.86), and *depression* (8 items, *α* = 0.90). Therefore, eating disorders (5 items, *α* = 0.70) and self-harm (3 items, *α* = 0.72) were excluded. Among externalizing mental health problems, we investigated those with “excellent” (≥0.90) or “good” (≥80 to < 90) Cronbach’s alphas (*α*) and diagnoses that were included in the DSM-5: *ADHD* (16 items, *α* = 0.89), *conduct disorder* (16 items, *α* = 0.95) and *oppositional defiant disorder* (9 items, *α* = 0.84) were used. For each mental health problem, a continuous score was used.

### Covariates

The following covariates are known to be associated with childhood sleep dimensions and/or mental health problems in adolescence [18–27]:

### Covariates during infancy

- Child’s sex—girls/ boys;
- Low maternal education when the child was 5 months of age (not having a high school diploma versus having a high school diploma);
- Maternal immigrant status when the child was 5 months of age—yes/ no (nonimmigrant);
- Familial income was measured when the child was 5 months old based on three indicators: family income, family size, and geographic area based on national census data—sufficient/ insufficient income.
- Childhood behavioral problems were measured when the child was 29 months of age: Hyperactivity-inattention (7 items), anxiety (3 items), aggression (13 items), and opposition (3 items). Mothers had to determine how often their child manifested each behavioral item in the past 3 months: “never,” “sometimes” or “often.” The Institut de la statistique du Québec standardized all behavioral variables on a scale from 0 (“not at all”) to 10 (“exactly”). These behavioral scales were used as continuous variables and had previously been shown to have good consistency with the Social Behavioral Questionnaire [28].

### Covariates in adolescence

- Meantime in bed during the week was self-assessed at age 15. Meantime in bed during the week was defined as time from bedtime to waketime during weekdays and weekends: (Time in bed during the weekdays*5) + (Time in bed during the weekend*2)/ 7.
- Waking difficulties were self-assessed at age 15 with one question: “How often do you have difficulty to wake up?.” Answers were categorized as “never,” “rarely,” “seldom,” “often,” and “always.” Again, we recoded the answers into 1 (never too often) and 2 (always).
- Sleepiness in class and/or while doing homework was self-assessed at age 15 with two questions: “How often do you fall asleep or you are drowsy in class?” and “How often do you fall asleep or you are drowsy during your homework?.” Answers were categorized as “never,” “rarely,” “seldom,” “often,” and “always.” We recoded responses into 1 (never to seldom) and 2 (often and always).

## Statistical Analyses

### Childhood sleep dimensions trajectories

The present work is based on three childhood sleep dimensions trajectories: (1) nighttime sleep duration (sleep quantity) measured at 2.5, 3.5, 4, 6, 8, 10, and 12 years of age, (2) sleep latency (difficulties in initiating sleep) measured at 2.5, 3.5, 4, 6, 8, and 10 years of age, and (3) WASO (difficulties in maintaining sleep) measured at 2.5, 3.5, 4, 6, 8, and 10 years of age. Up to three missing data points per participant were permitted. Trajectory models allowed for 2 to 5 trajectories of various shapes (intercept -0-, linear -1-, quadratic -2-, or cubic -3-) [22]. Several indicators of fit exist to determine the best model. The Bayesian Information Criterion and Akaike Information Criterion (AIC) were employed to determine the optimal number and shape of trajectories that best fitted the data, and then each participant was assigned to a specific sleep trajectory based on the highest posterior probability of belonging to one specific trajectory. As the average group posterior probability (AvePP) approaches 1, it means that the model fits better. An AvePP greater than 0.70 for all groups is recommended. It is also important to verify the odds of correct classification for each trajectory. For a model that fits the data well, odds of correct classification values should be much greater than 1. Finally, when a model fits well, the difference between estimated group probabilities and the proportion assigned to the group using the maximum probability rule (|π-*p*|) should be near zero because these two quantities should be quite similar. The childhood sleep dimensions trajectories were computed using the PROC TRAJ procedure in SAS, version 9.1.

Using SPSS 28, chi-squared tests were conducted with childhood sleep trajectories and child’s sex to examine whether the childhood sleep trajectories are the same for boys and girls. Descriptive statistics were computed to describe all variables in the initial sample and Pearson correlations were used to examine associations between adolescent mental health outcomes and childhood sleep dimensions. A path analysis model was assessed in the full sample using Mplus statistical software version 8.2. Interaction terms child’s sex × childhood sleep trajectories were removed to obtain a more parsimonious model because they were nonsignificant. At age 15, 1446 adolescents (65.0% of the sample) filled out the Mental Health and Social Inadaptation Assessment for Adolescents. Missing data due to attrition were handled with full information maximum likelihood, which uses maximum likelihood to estimate model parameters using all available raw data [29, 30]. Based on recommended guidelines [31, 32], overall model fit was tested with the listwise deletion model using the following indicators of fit: comparative fit index (CFI), root mean square error of approximation (RMSEA), standardized root mean square residual (SRMR), and the ratio of chi-square to degrees of freedom (χ^2^/*df*). A CFI value of 0.90 or higher, a RMSEA and a SRMR value below 0.05, and a χ^2^/*df* less than 3.00 are indicators of good fit. All statistical analyses were two-tailed and the significance level was set at *p*-value < 0.05.

## Results

### Childhood sleep trajectories

**Figure 1.**
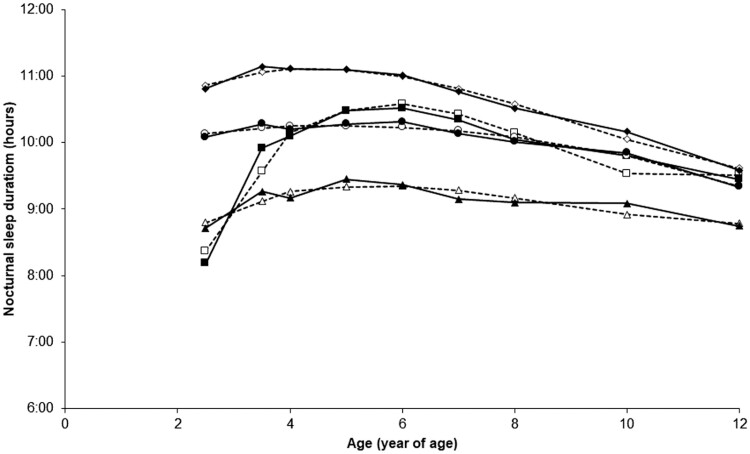
Four childhood nighttime sleep duration trajectories from 2.5 to 12 years of age were obtained: ▲: children following the short-persistent trajectory (7.5%, *n* = 100), ■: children following the short-increasing trajectory (5.8%, *n* = 77), ●: children following the 10-hour trajectory (50.7%, *n* = 677), and ♦: children following the 11-hour trajectory (36.0%, *n* = 481). Source. Data were compiled from the final master file of the Québec Longitudinal Study of Child Development (1998–2013), ©Gouvernement du Québec, Institut de la statistique du Québec.

For *childhood nighttime sleep duration trajectories*, a four-trajectory solution with a “3 3 2 3” profile (three cubic and one quadratic trajectories) was identified as the best-fitting model (BIC = -12758.73, AIC = −12709.36, *n *= 1335, censored normal). Figure 1 illustrates the four trajectories that best-fit childhood nocturnal sleep duration courses from ages 2.5 to 12 years: (1) a short-persistent pattern (7.5%, *n *= 100) composed of children sleeping less than 10 hours per night until age 12; (2) a short-increasing pattern (5.8%, *n *= 77) composed of children who slept fewer hours in early childhood but whose sleep duration increased around age 4; (3) a 10-hour persistent pattern (50.7%, *n *= 677) composed of children who persistently slept around 10 hours per night from 2.5 up to 12 years of age; (4) an 11-hour declining pattern (36.0%, *n *= 481) composed of children who slept around 11 hours per night in early childhood declining to about 10 hours of sleep later on. The average group posterior probability estimates were 0.89 for the short sleep duration persistent trajectory (OCC = 96.7, |π-*p*|=0.005), 0.79 for the short-increasing duration trajectory (OCC = 51.1, |π-*p*|=0.013), 0.85 for the 10-hour persistent sleep duration trajectory (OCC = 6.1, |π-*p*|=0.018), and 0.88 for the 11-hour-declining sleep duration trajectory (OCC = 12.9, |π-*p*|=0.000). There was no difference in the proportion of girls and boys in *nighttime sleep duration trajectories* (χ^2^ = 6.41, *df* = 3, *p* = 0.09).

**Figure 2.**
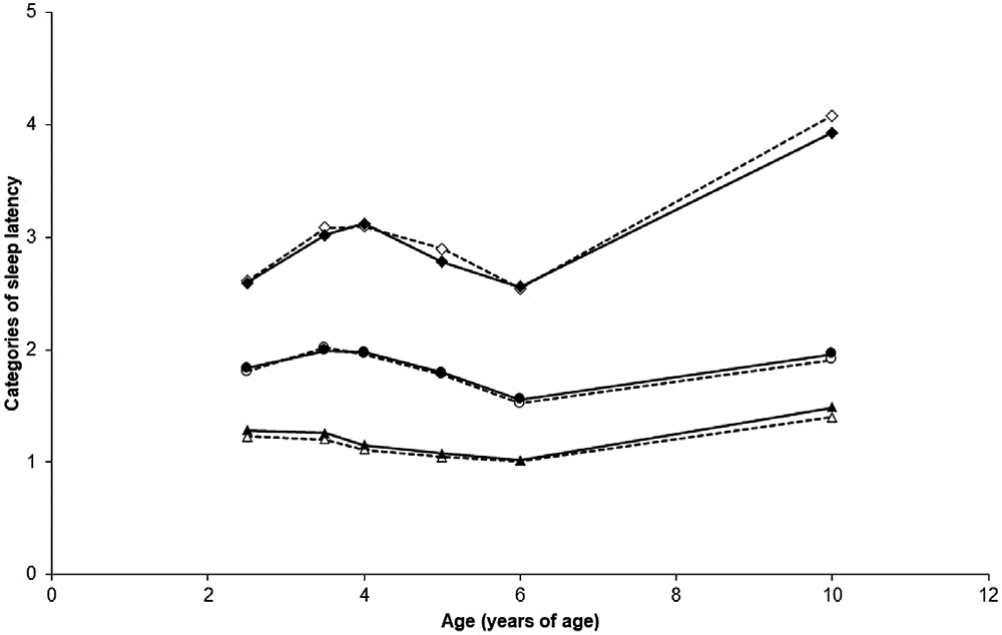
Three childhood sleep latency trajectories from 2.5 to 10 years of age were obtained: ▲: children following the short sleep latency trajectory (31.7%, *n* = 510), ●: children following the intermediate sleep latency trajectory (59.9%, *n* = 963) and ♦: children following the long sleep latency trajectory (8.4%, *n* = 135). Response categories were: (1) < than 15 minutes, (2) from 15 minutes to < than 30 minutes, (3) from 30 minutes to < 45 minutes, (4) from 45 minutes to < than 60 minutes, and (5) ≥ 60 minutes. Source: Data were compiled from the final master file of the Québec Longitudinal Study of Child Development (1998–2013), ©Gouvernement du Québec, Institut de la statistique du Québec.

For *childhood sleep latency trajectories*, a three-trajectory solution with a ‘3 3 3’ profile (three cubic trajectories) was identified as the best-fitting model (BIC = −10428.56, AIC = −10388.19, *n* = 1608, censored normal). Figure 2 draws the three trajectories that best-fit childhood sleep latency courses from ages 2.5 to 10 years: (1) a short sleep latency pattern (31.7%, *n *= 510) composed of children taking less than 15 minutes to fall asleep; (2) an intermediate sleep latency pattern (59.9%, *n* = 963) composed of children taking between 15 and 30 minutes to fall asleep and (3) a long sleep latency pattern (8.4%, *n *= 135) composed of children taking between 30 and 60 minutes to fall asleep between the ages of 2.5 and 10 years. The average group posterior probability estimates were 0.88 for the short sleep latency trajectory (OCC = 16.2, |π-*p*|=0.000), 0.90 for the intermediate sleep latency trajectory (OCC = 6.4, |π-*p*|=0.012) and 0.88 for the long sleep latency trajectory (OCC = 68.7, |π-*p*|=0.012). There was no difference in the proportion of girls and boys in *sleep latency trajectories* (χ^2^ = 1.16, *df* = 1, *p = *0.56).

**Figure 3.**
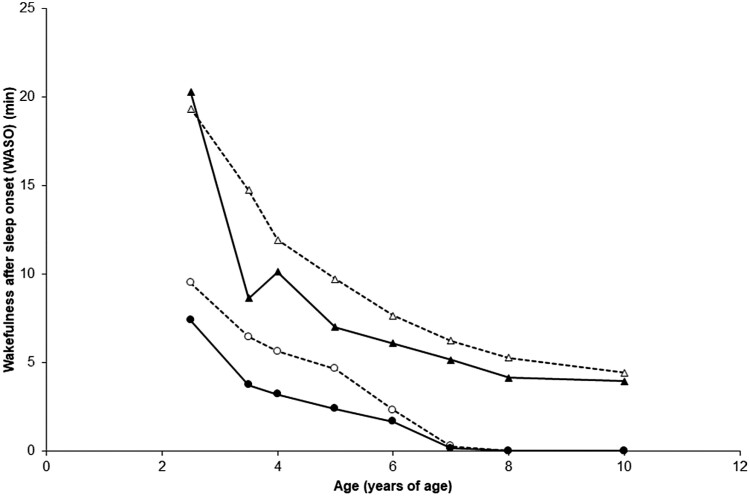
Two wakefulness after sleep-onset (WASO) trajectories from 2.5 to 10 years of age were obtained: ●: children following the normative WASO trajectory (73.0%, *n* = 1026) and ▲: children following the long WASO trajectory (23.0%, *n* = 379). Source: Data were compiled from the final master file of the Québec Longitudinal Study of Child Development (1998–2013), ©Gouvernement du Québec, Institut de la statistique du Québec.

For *childhood WASO trajectories*, a two-trajectory solution with a “3 2” profile (1 cubic and 1 quadratic trajectories) was identified as the best-fitting model (BIC = -4330.47, AIC = -4306.86, *n* = 1405, censored normal). Figure 3 depicts the 2 trajectories that best-fitted childhood nocturnal sleep duration courses from ages 2.5 to 10 years: (1) a normative WASO trajectory (73.0%, *n *= 1026) composed of children who did not wake up during the night since age 7 and (2) a long WASO trajectory (27.0%, *n *= 379). The average group posterior probability estimates were 0.87 for the normative WASO trajectory (OCC = 3.5, |π-*p*|=0.064) and 0.90 for the long WASO trajectory (OCC = 18.1, |π-*p*|=0.064). There was no difference in the proportion of girls or boys in WASO *trajectories* (χ^2^ = 1.49, *df* = 1, *p* = 0.22).

### Adolescent mental health outcomes and childhood sleep trajectories

Table 1 presents all descriptive data on the initial sample. Pearson correlations among adolescent mental health outcomes and sleep dimensions are reported in Table 2. Social phobia, generalized anxiety, depression, ADHD, conduct problems, and opposition were modestly related (from *r* = 0.15 to *r* = 0.62); this corroborates to use of a path analysis model allowing residual terms of dependent variables to covary. However, childhood sleep trajectories were weekly related (*r* = −0.10 to *r* = 0.12); this corroborates the examination of the three types of childhood sleep trajectories in relation to adolescent mental health outcomes.

**Table 1.** Descriptive Data on Adolescent Mental Health Outcomes, Childhood Sleep Trajectories and Covariates (*n* = 2120)

	Frequency§	%§ or SDǁ	Missing values (*n*)
Characteristics	or meanǁ		
*Adolescent mental health outcomes (15 y)*			
Social phobiaǁ	2.40	2.18	780
Generalized anxietyǁ	4.10	2.17	780
Depressionǁ	3.89	2.25	781
ADHDǁ	2.90	1.68	783
Conduct problemsǁ	0.65	0.90	782
Oppositionǁ	2.35	1.39	782
*Childhood sleep trajectories (from 2.5 to 10/12 y)*			
Nocturnal sleep duration trajectories§			888
Short sleep duration trajectory	100	7.5	
Short-increasing sleep duration trajectory	77	5.8	
10-11 h sleep duration trajectory	1158	86.7	
Latency sleep-onset trajectories§			615
Long sleep-onset trajectory	135	8.4	
Intermediate sleep-onset trajectory	963	59.9	
Short sleep-onset trajectory	510	31.7	
Wakefulness after sleep-onset trajectories§			818
Long WASO trajectory	379	27.0	
Normative WASO trajectory	1026	73.0	
*Covariates*			
Child’s sex§			0
Boys	1138	51.2	
Girls	1085	48.8	
Maternal education§			3
Having a high school diploma	1819	81.9	
Not having a high school diploma	401	18.1	
Maternal immigrant status§			2
Yes	260	11.7	
No	1961	88.3	
Familial income§			41
Sufficient	1657	75.9	
Not sufficient	525	24.1	
Child hyperactivity-inattention at 2.5 yǁ	3.49	2.15	226
Child anxiety at 2.5 yǁ	1.03	1.54	227
Child aggressivity at 2.5 yǁ	1.75	1.40	226
Child opposition at 2.5 yǁ	3.6	2.29	226
Number of sleep awakenings (at 2.5 y)§			330
No awakening	497	26.3	
Less than 1 per night	662	35.0	
1–2 awakenings per night	495	26.1	
3–4 awakenings per night	140	7.4	
5 or more awakenings per night	99	5.2	
Mean time in bed during the week at age 15ǁ	9.02	0.91	847
Waking difficulties at age 15§			800
Always	248	17.4	
No	1175	82.6	
Sleepiness in class/doing homework at age 15§			798
Yes	267	18.7	
No	1158	81.3	

Source: Data were compiled from the final master file of the Québec Longitudinal Study of Child Development (1998–2013), ©Gouvernement du Québec, Institut de la statistique du Québec.

^§^means categorical variables.

^ǁ^means continuous variables.

**Table 2.** Pearson Correlations Between Adolescent Mental Health Outcomes and Childhood Sleep Dimensions (*n* = 1782)

Variables	1	2	3	4	5	6	7	8	9	10	11	12	13	14	15
1. Social phobia (15 y)															
2. Generalized anxiety (15 y)	0.55**														
3. Depression (15 y)	0.52**	0.74**													
4. ADHD (15 y)	0.31**	0.49**	0.59**												
5. Conduct problems (15 y)	0.15**	0.26**	0.37**	0.54**											
6. Opposition (15 y)	0.20**	0.31**	0.43**	0.62**	0.59**										
7. Short sleep persistent trajectory (2.5 to 12 y)	0.05	0.01	0.00	−0.02	−0.01	*−0.06									
8. Short sleep-increasing trajectory (2.5 to 12 y)	0.02	0.02	0.01	0.03	0.01	0.05	*−0.07								
9. 10 h sleep duration trajectories (2.5 to 12 y)	−0.04	−0.01	0.01	−0.02	−0.01	−0.04	**−0.29	**−0.25							
10. 11 h sleep duration trajectories (2.5 to 12 y)	0.00	−0.01	−0.01	0.02	0.01	0.05	**−0.21	**−0.19	**−0.76						
11. Short sleep latency trajectory (2.5 to 10 y)	−0.05	−0.01	−0.04	−0.05	*−0.07	*−0.06	−0.05	*−0.06	−0.04	0.09**					
12. Intermediate sleep latency trajectory (2.5 to 10 y)	0.05	0.01	0.00	0.01	0.05	0.02	−0.02	0.05	0.03	−0.04	**−0.83				
13. Long sleep latency trajectory (2.5 to 10 y)	−0.01	0.00	0.06*	0.06*	0.04	0.07*	0.12**	0.02	0.01	**−0.08	**−0.21	**−0.37			
14. Number of sleep awakenings (2.5 y)	0.01	0.01	0.01	−0.01	−0.02	−0.02	0.12**	0.05	−0.01	**−0.08	−0.05	0.05	0.01		
15. Normal WASO trajectory (2.5 to 10 y)	−0.04	−0.01	0.01	0.03	0.05	0.05	**−0.10	−0.03	−0.03	0.10**	0.07**	−0.03	*−0.06	**−0.20	
16. Long WASO trajectory (2.5 to 10 y)	0.04	0.01	−0.01	−0.03	−0.05	−0.05	0.10**	0.03	0.03	**−0.10	**−0.07	0.03	0.06*	0.20**	**−1.00

* *p* < 0.05, ** *p *< 0.01.

Source: Data were compiled from the final master file of the Québec Longitudinal Study of Child Development (1998–2013), ©Gouvernement du Québec, Institut de la statistique du Québec..

### Associations between childhood sleep trajectories and adolescent mental health outcomes

Table 3 shows the results of a path analysis model. The model shows good fit: χ^2^(96) = 134.78, *p* = 0.006; RMSEA = 0.015, 90% CI: 0.008 to 0.021; CFI = 0.990; SRMR = 0.021; and χ^2^(*df*) = 1.40.

**Table 3.** Results of a Path Analysis Examining Adolescent Mental Health Outcomes at 15 Years of Age as a Function of Childhood Sleep Trajectories After Adjusting on Sociodemographic Variables, Behavioral Problems at 2.5 Years of Age and Concomitant Sleep Dimensions at 15 Years (*n* = 1782)

	Social phobia		Generalized anxiety		Depression		ADHD		Conduct problems		Opposition	
	*β*	*P*	*β*	*P*	*β*	*P*	*β*	*P*	*β*	*P*	*β*	*P*
*Childhood sleep trajectories*												
Nocturnal sleep duration, 10/11 h (ref)												
Short sleep duration	0.05	0.15	0.02	0.46	0.00	0.87	−0.03	0.23	−0.04	0.18	**−0.08**	**0.003**
Short-increasing sleep duration	0.03	0.36	0.03	0.3	0.02	0.40	0.02	0.52	−0.002	0.92	0.03	0.25
Latency to sleep-onset, short sleep onset (ref)												
Intermediate sleep onset	0.04	0.16	0.005	0.85	0.02	0.40	0.03	0.27	**0.06**	**0.04**	0.04	0.17
Long sleep onset	−0.01	0.67	−0.01	0.74	**0.06**	**0.02**	**0.07**	**0.01**	**0.05**	**0.05**	**0.08**	**0.010**
Wakefulness after sleep onset (WASO), normal (ref)												
Long WASO	0.02	0.48	−0.01	0.63	−0.04	0.15	−0.04	0.20	**−0.06**	**0.03**	**−0.06**	**0.04**
Number of sleep awakenings (at 2.5 y)	−0.005	0.86	0.004	0.87	0.006	0.80	−0.006	0.83	−0.02	0.57	−0.01	0.60
Sociodemographic variables												
Child’s sex, boys (ref)	**0.23**	**<.001**	**0.34**	**<.001**	**0.34**	**<.001**	0.03	0.18	0.007	0.78	−0.01	0.65
Low maternal education	−0.01	0.6	0.00	0.96	−0.01	0.56	0.00	0.90	0.05	0.08	0.05	0.08
Maternal immigrant status	0.03	0.26	0.02	0.37	0.02	0.44	0.00	0.98	0.02	0.55	−0.01	0.64
Insufficient familial income	−0.02	0.57	−0.02	0.38	−0.01	0.59	0.04	0.18	0.06	0.06	**0.07**	**0.01**
Behavioral problems (at 2.5 y)												
Child hyperactivity-inattention	0.05	0.14	0.06	0.05	0.03	0.23	**0.09**	**0.003**	0.02	0.44	**0.07**	**0.02**
Child anxiety	0.01	0.70	0.005	0.85	−0.04	0.10	**−0.05**	**0.04**	**−0.06**	**0.01**	−0.04	0.17
Child aggression	−0.003	0.92	−0.03	0.36	0.00	0.96	0.05	0.15	0.06	0.11	**0.06**	**<0.05**
Child opposition	0.04	0.22	0.00	0.88	0.01	0.66	−0.01	0.64	0.04	0.23	0.000	0.99
Sleep dimensions (at age 15 y)												
Mean time in bed during the week	−0.02	0.41	−0.05	0.06	**−0.07**	**0.02**	**−0.06**	**0.02**	**−0.15**	**<.001**	**−0.08**	**0.01**
Waking difficulties	0.05	0.08	**0.09**	**0.001**	**0.11**	**<.001**	**0.12**	**<.001**	**0.10**	**0.002**	**0.15**	**<.001**
Sleepiness in class/ while doing homework	**0.11**	**<.001**	**0.19**	**<.001**	**0.27**	**<.001**	**0.25**	**<.001**	**0.17**	**<.001**	**0.16**	**<.001**
R squared	0.09		0.20		0.25		0.13		0.10		0.11	

Data were compiled from the final master file of the Québec Longitudinal Study of Child Development (1998–2013), ©Gouvernement du Québec, Institut de la statistique du Québec.

Values in bold mean significant results (P<0.05).

### Social phobia

The model indicates that none of the childhood sleep trajectories was associated with symptoms of social phobia in adolescence. Not surprisingly, being a girl is positively associated with symptoms of social phobia (*β* = 0.23, 95% CI: 0.19 to 0.28). Finally, sleepiness in class or/and while doing homework at age 15 is positively associated with symptoms of social phobia (*β* = 0.11, 95% CI: 0.05 to 0.17).

### Generalized anxiety

The model shows that none of the childhood sleep trajectories was associated with symptoms of generalized anxiety in adolescence. Also, being a girl is positively associated with symptoms of generalized anxiety (*β* = 0.34, 95% CI: 0.30 to 0.39). Among concomitant sleep dimensions, waking difficulties at age 15 are positively associated with symptoms of generalized anxiety in adolescence (*β* = 0.09, 95% CI: 0.04 to 0.14). Finally, sleepiness in class or/and while doing homework at age 15 is positively associated with symptoms of generalized anxiety in adolescence (*β* = 0.19, 95% CI: 0.14 to 0.24).

### Depression

Compared with the short sleep latency, a long childhood sleep latency trajectory predicted a higher depression score in adolescence (*β* = 0.06, 95% CI: 0.01 to 0.12). Also, being a girl is positively associated with depressive symptoms in adolescence (*β* = 0.34, 95% CI: 0.29 to 0.38). Among concomitant sleep dimensions, an average time in bed during the week at age 15 is negatively associated with depressive symptoms in adolescence (*β* = −0.07, 95% CI: −0.12 to −0.01). Waking difficulties at age 15 are positively associated with depressive symptoms in adolescence (*β* = 0.11, 95% CI: 0.06 to 0.16). Finally, sleepiness in class or/and while doing homework at age 15 is positively associated with depressive symptoms in adolescence (*β* = 0.27, 95% CI: 0.22 to 0.32).

### Attention deficit hyperactivity disorder

Compared with the short sleep latency, a long childhood sleep latency trajectory predicted a higher ADHD score in adolescence (*β* = 0.07, 95% CI: 0.02 to 0.13). Among behavioral problems at 2.5 years of age, children’s hyperactivity-inattention reported by mothers predicted a higher ADHD score in adolescence (*β* = 0.09, 95% CI: 0.03 to 0.10). Conversely, children’s anxiety reported by mothers at 2.5 years of age predicted a higher ADHD score in adolescence (*β* = −0.05, 95% CI: −0.10 to −0.002). Among concomitant sleep dimensions, an average time in bed during the week at age 15 is negatively associated with ADHD symptoms in adolescence (*β* = −0.06, 95% CI: −0.12 to −0.01). Waking difficulties at age 15 are positively associated with ADHD symptoms in adolescence (*β* = 0.12, 95% CI: 0.07 to 0.18). Finally, sleepiness in class or/and while doing homework at age 15 is positively associated with ADHD symptoms in adolescence (*β* = 0.25, 95% CI: 0.19 to 0.30).

### Conduct problems

Compared with the short sleep latency, a long childhood sleep latency trajectory predicted a higher score of conduct problems in adolescence (*β* = 0.05, 95% CI: 0.00 to 0.10). In addition, compared with the short sleep latency, an intermediate childhood sleep latency trajectory predicted a higher score on conduct problems in adolescence (*β* = 0.06, 95% CI: 0.003 to 0.11). Finally, a long childhood WASO is negatively associated with conduct problems in adolescence (*β* = −0.06, 95% CI: −0.11 to −0.01). Children’s anxiety reported by mothers at 2.5 years of age predicted a higher score on conduct problems in adolescence (*β* = −0.06, 95% CI: −0.10 to −0.02). Among concomitant sleep dimensions, an average time in bed during the week at age 15 is negatively associated with symptoms of conduct problems in adolescence (*β* = −0.15, 95% CI: −0.22 to −0.08). Waking difficulties at age 15 are positively associated with symptoms of conduct problems in adolescence (*β* = 0.10, 95% CI: 0.03 to 0.16). Finally, sleepiness in class or/and while doing homework at age 15 is positively associated with symptoms of conduct problems in adolescence (*β* = 0.17, 95% CI: 0.11 to 0.23).

### Opposition

Compared with the short sleep latency, a long childhood sleep latency trajectory predicted a higher opposition score in adolescence (*β* = 0.08, 95% CI: 0.02 to 0.13). Compared with 10-11h sleep duration trajectories, a short sleep duration trajectory is negatively associated with opposition in adolescence (*β* = −0.08, 95% CI: −0.14 to −0.03). Finally, a long childhood WASO is negatively associated with opposition in adolescence (*β* = −0.06, 95% CI: −0.11 to −0.03). Among sociodemographic variables, an insufficient familial income is positively associated with opposition in adolescence (*β* = 0.07, 95% CI: 0.02 to 0.13). Among behavioral problems at 2.5 years of age, children’s hyperactivity-inattention reported by mothers predicted a higher opposition score in adolescence (*β* = 0.07, 95% CI: 0.02 to 0.13). Also, children’s aggression reported by mothers at 2.5 years of age predicted a higher opposition score in adolescence (*β* = 0.06, 95% CI: 0.001 to 0.13). Among concomitant sleep dimensions, an average time in bed during the week at age 15 is negatively associated with symptoms of opposition in adolescence (*β* = −0.08, 95% CI: −0.13 to −0.02). Waking difficulties at age 15 are positively associated with symptoms of opposition in adolescence (*β* = 0.15, 95% CI: 0.09 to 0.21). Finally, sleepiness in class or/and while doing homework at age 15 is positively associated with symptoms of opposition in adolescence (*β* = 0.16, 95% CI: 0.11 to 0.22).

## Discussion

The present study aimed to assess whether multiple facets of sleep (nocturnal sleep duration, sleep latency, WASO) were associated with adolescent symptoms of DSM-5-defined mental health outcomes, namely social phobia, generalized anxiety, depressive, ADHD, conduct problems and opposition at age 15 while controlling for covariates (sociodemographic variables, early childhood behavioral problems, and concomitant sleep dimensions). The main results of the model indicated that following a long childhood sleep latency trajectory predicted higher scores on depression, ADHD, conduct disorders, and opposition at age 15 after controlling for the above covariates compared with a trajectory of short sleep latency.

### Long childhood sleep latency trajectory and adolescent mental health outcomes

It was hypothesized that children following a long sleep latency trajectory throughout childhood would be at higher risk of poor mental health outcomes in adolescence. The results partially support our hypothesis for symptoms of depression, ADHD, conduct disorder, and opposition (not for symptoms of social phobia and generalized anxiety). Different underlying mechanisms could explain these associations according to different types of mental health outcomes. For depression, an internalizing problem, this could suggest that impairments of neurotransmitter systems from early childhood (e.g. GABAergic transmission and serotonergic system) could have an effect on sleep regulation and possibly, through a looping rumination mechanism, increasing the risk of developing depression later [12]. Indeed, Marino and her colleagues [33] found bidirectional relationships between disturbed sleep and depressive symptoms throughout childhood up to 13 years of age, which suggests that disturbed sleep makes a significant contribution to the persistence of depressive symptoms across childhood. For ADHD, conduct problems and opposition, which are defined as externalizing problems, the association with long latency to sleep could be due to neurological aspects and parental-child factors, such as bedtime resistance. It is well known that symptoms of ADHD could lead to persistent movements and could probably contribute to explain why these children took more time to fall asleep in childhood. In addition, childhood bedtime resistance, a form of protodyssomnia [8], increases sleep latency and is a form of opposition to parental guidance toward sleep. To the best of our knowledge, this is the first study that links childhood sleep latency (difficulty of initiating sleep) with conduct problems and opposition in adolescence; this should be verified in future studies.

### Short nocturnal sleep duration and adolescent mental health outcomes

It was hypothesized that children following a short nocturnal sleep duration trajectory throughout childhood would be at higher risk of poor mental health outcomes in adolescence. The results did not support our hypothesis. Conversely, we found that a short sleep duration trajectory compared with 10–11 hours sleep duration trajectories is negatively associated with opposition in adolescence. To the best of our knowledge, this is the first study that links childhood nocturnal sleep duration with opposition in adolescence; this result should be verified in future studies with actigraphic recordings in order to reduce potentially maternal bias.

### Long wakefulness after sleep onset and adolescent mental health outcomes

It was hypothesized that children following a long wakefulness after sleep-onset trajectory throughout childhood would be at higher risk of poor mental health outcomes in adolescence. The results did not support our hypothesis. Conversely, we found that a long childhood WASO is negatively associated with conduct problems and opposition in adolescence. This may be explained that parents were less accurate in reporting WASO because their children potentially signaled less their sleep awakenings to their parents. As shown by another study [34], preschoolers involved in Child Protective Services signaled significantly fewer nocturnal sleep awakenings to their parents compared to the group of preschoolers from the community. This result should be corroborated by future longitudinal studies with actigraphic recordings.

### Strengths and limitations

The above findings must be interpreted in light of the present study’s strengths and weaknesses. The analyses benefited from the use of a large, prospective, longitudinal sample, allowing the observation of relationships across developmental periods. However, nighttime sleep duration was assessed through maternal reports, which may lead to an overestimation of sleep duration due to the lack of information on the time taken to fall asleep, or the number and length of nocturnal awakenings. Nevertheless, previous studies have shown a good correlation between nocturnal sleep duration reported by parents and actigraphy measures [35]. However, parental reports of wakefulness after sleep onset are subjected to bias because it is not all children who signal their nocturnal awakenings to their parents. Actigraphy could be a solution to evaluate more precisely childhood sleep dimensions (e.g. sleep duration, sleep latency, and WASO) but actigraphy also has its own limitations such as low specificity in detecting wakefulness [36]. In addition, mental health assessment relied on self-reports; this is the most popular method for collecting mental health information in adolescents in population samples, this is the most popular method for collecting mental health information in adolescents but it cannot replace assessments performed by a clinician. In addition, we did not assess the role of pubertal status, which is known to be linked with both sleep and mental health. Despite its limitations, this study provides evidence that a long childhood sleep latency predicted higher scores on depression, ADHD, conduct disorders, and opposition at age 15 compared to shorter one. These data should be replicated with objective measures.

## Conclusion

In conclusion, the results of this longitudinal study revealed for the first time that children presenting a long childhood sleep latency throughout childhood (difficulties in initiating sleep) could be at higher risk of developing mental health problems later on in adolescence. This could lead to better promote preventive sleep interventions during childhood in order to reduce the risk of developing mental health problems in adolescence. These associations are weak but remain statistically significant after adjusting for covariates in a population sample. These results should be replicated in longitudinal clinical samples. Further longitudinal investigations incorporating other lifestyle behaviors including poor diet, poor physical activity, excessive recreational screen time, and parental styles would help to clarify which factors could explain why children presenting a long childhood sleep latency are at greater risk of symptoms of depression, ADHD, conduct problems and opposition in adolescence.

## Notes

Submitted to: ***Sleep Advances***
